# “Close your eyes and relax”: the role of hypnosis in reducing anxiety, and its implications for the prevention of cardiovascular diseases

**DOI:** 10.3389/fpsyg.2024.1411835

**Published:** 2024-07-05

**Authors:** Donato Giuseppe Leo, Simon S. Keller, Riccardo Proietti

**Affiliations:** ^1^Department of Cardiovascular and Metabolic Medicine, Institute of Life Course and Medical Sciences, Faculty of Health and Life Science, University of Liverpool, Liverpool, United Kingdom; ^2^Liverpool Centre for Cardiovascular Science, University of Liverpool and Liverpool Heart and Chest Hospital, Liverpool, United Kingdom; ^3^Department of Pharmacology and Therapeutics, Institute of Systems, Molecular and Integrative Biology, Faculty of Health and Life Sciences, University of Liverpool, Liverpool, United Kingdom

**Keywords:** anxiety, cardiovascular disease, hypnosis, hypnotherapy, mental health

## Abstract

Anxiety is the most common form of mental health disorder, affecting millions of people worldwide. Psychosocial interventions such as mindfulness and cognitive behavioral therapy (CBT) have been suggested as an effective treatment in the management of general anxiety and anxiety disorders, with emerging evidence also suggesting the effectiveness of hypnosis. Moreover, anxiety has shown to be linked to the onset and development of several cardiovascular diseases (CVD), which are the leading cause of global death. In this paper, we review the current literature to examine the role that anxiety has on the onset and development of CVD and summarize the current knowledge on the role that hypnosis and hypnotherapy have in reducing anxiety, also explaining how this can impact the cardiovascular system and the prevention of CVD. Review of the evidence suggests that hypnosis and hypnotherapy are effective in treating anxiety and may positively affect the heart and the cardiovascular system, reducing sympathetic activation and increasing parasympathetic tone, potentially preventing the onset of CVD related to increased sympathetic activation. However, further studies are required to further understand how hypnosis and hypnotherapy affect the cardiovascular system through investigation of the neurophysiological components of the hypnotic state and of the mind-body relationship. Healthcare systems should embed mental health screening in patients at risk of developing CVD as part of the clinical pathway and consider the role that hypnosis and hypnotherapy may play in the management of CVD.

## Introduction

Anxiety is defined as an emotion characterized by feelings of dread and internal turmoil due to anticipated events seen as menacing to the self (Arroll and Kendrick, 2018). Anxiety disorders (e.g., phobias, post-traumatic stress disorder – PTSD) evolve from the general and normal emotion of fear, to the point of impacting the affected person’s life to different extents (Rosen and Schulkin, 1998; American Psychiatric Association [APA], 2015). Anxiety can have various manifestations (American Psychiatric Association [APA], 2015), such as “separation anxiety” (abnormal stress about the thought of being away from home or loved ones), “performance anxiety” (e.g., athletic performance anxiety, stage fright, test-taking anxiety), or “social anxiety” (abnormal worrying of social situations, such as being in public, meeting new people, speaking to the phone, have social conversations). Anxiety can be an acute state or a long-term characteristic of an individual, and may induce somatic symptoms, with women reporting a higher prevalence in its development compared to men (Bekker and van Mens-Verhulst, 2007). Around 4% of the global population suffers from at least one anxiety disorder (WHO, 2023a), making it the most common mental health disorder worldwide. Risk factors for developing anxiety may be genetic (Michael et al., 2007; Shimada-Sugimoto et al., 2015) (neurochemical dysfunction involving autonomic imbalance, increased cortisol production, increased function of the adenosine receptor, or decreased GABA-ergic tone), psychological (Donovan and Spence, 2000; Michael et al., 2007) (behavioral inhibition, negative life events, transfer of parental anxiety), social (Jones et al., 2017) (bullying, gender socialization), environmental (Michael et al., 2007; American Psychiatric Association [APA], 2015) (drug use, alcohol and high caffeine consumption), or associated to medical conditions (Muller et al., 2005) (such as chronic obstructive pulmonary disease - COPD, endocrine diseases, cardiovascular diseases, brain degenerative diseases). Symptoms varies depending on the type of anxiety, with the most common symptoms including acute panic attacks, headache, nausea, diarrhea, frequent urination, sexual dysfunctions, palpitation, shortness of breath, increased skin perspiration, paresthesia, and tremors (Testa et al., 2013; American Psychiatric Association [APA], 2015). Anxiety can induce depression (Kalin, 2020) or other mental disorders (Lewinsohn et al., 2008), also leading to self-harm and suicide (Kanwar et al., 2013). The diagnosis of anxiety disorder accounts for the persistent manifestation of symptoms (typically lasting 6 months or more) experienced by the person, which deeply impact their daily living (Testa et al., 2013; Ströhle et al., 2018). Treatment is tailored to the type of anxiety (Bandelow et al., 2017), and can include psychological interventions (e.g., mindfulness, cognitive behavioral therapy – CBT), pharmacological interventions (e.g., antidepressants), or a combination of the two.

Cardiovascular diseases (CVD) are a class of health conditions that affect the heart and the blood vessels (Joseph et al., 2017), such as arrhythmias, heart failure, hypertension, coronary artery disease, etc. CVDs are considered the leading cause of death worldwide, with 17.9 million deaths per year (WHO, 2023b). Common risk factors for the development of CVD are age, sex, smoking, alcohol consumption, diet, sedentary behaviors, and genetic predisposition (Joseph et al., 2017). Anxiety and emotional stress have been shown to play an important role in the development of CVD (Silverman et al., 2019). Chronic stress influences the hypothalamus-pituitary-adrenal axis (HPA) activation that leads to increased heart rate and blood pressure, and to endothelial dysfunction that can trigger atherosclerosis (Lagraauw et al., 2015). Phobias have been linked to an increased risk of coronary artery disease (Emdin et al., 2016), and PTSD has been linked to an increased risk of stroke (Emdin et al., 2016). A recent article has also suggested the role that emotional stress may have in triggering cardiac arrhythmias (Leo et al., 2023). However, prevention and clinical management of CVD do not often consider the role that anxiety and emotional stress can play in the onset and progression of these conditions, missing a potential chance of improving the quality of life of patients with CVD.

Hypnosis is defined as a state of focused attention and reduced peripheral awareness that enhances the capacity of a person to respond to suggestion (Elkins et al., 2015). Hypnotherapy (the use of hypnosis as therapy) has been shown to be effective in treating anxiety (Valentine et al., 2019), and in reducing stress (Fisch et al., 2020), also increasing stress coping (Fisch et al., 2020). Despite the potential beneficial effects shown by hypnotherapy in treating anxiety and emotional stress, its uptake in the medical community is still low compared to well established psychological interventions, such as mindfulness and CBT. Therefore, the aims of this narrative review are to: (i) summarize the current literature on the effects that anxiety and emotional stress have on the onset and development of CVD, (ii) summarize the current literature on the role that hypnosis has in the treatment of anxiety and in the management of emotional stress, and (iii) discuss the role that hypnotherapy may have in the prevention and management of CVD thanks to its impact on the anxiety and stress level of patients.

## An anxious heart: effects of anxiety and emotional stress on the heart

Chronic stress can negatively affect the immune and endocrine system, also affecting the body’s metabolic response (Lagraauw et al., 2015). A crucial role in the regulation of the stress response is played by the HPA axis and the autonomic nervous system (Ziegler, 2012; Herman et al., 2016). Dysfunctions of the HPA axis have shown to increase stress vulnerability, also increasing the risk for stress-related mental disorders (Leistner and Menke, 2020). Animal models have shown that genetic factors are accountable for individual differences in the experience-dependent response to stress (Terenina et al., 2019). Additionally, chronic exposure to stress has shown to induce adaptation of the HPA (Herman and Tasker, 2016), potentially lowering the individual response to acute psychological stress (Matthews et al., 2001). Hyperactivity of the sympathetic nervous system induced by chronic stress (such as major depressive disorders), may lead to an increased activity of the immune system inducing a pro-inflammatory response (Won and Kim, 2016). Autonomic imbalance elicits by chronic stress, with increased sympathetic tone and reduced vagal tone, can lead to acute cardiovascular events (such as heart attack) (Hering et al., 2015).

Anxiety has been proposed as a possible independent risk factor for the onset of CVD (Suls, 2018; Karlsen et al., 2021). A prospective cohort study (Nakada et al., 2023) using UK Biobank and the data of 413,973 participants, showed that patients diagnosed with anxiety disorder had a higher risk of CVD (hazard ratio 1.72; 95% confidence interval: 1.32–2.24), which increased when combined with the diagnosis of depression (hazard ratio: 2.89; 95% confidence interval: 2.03–4.11). A population-based prospective cohort study (Vassou et al., 2024) conducted on 853 participants without CVD that completed psychological evaluation, showed that anxiety [assessed with the Irrational Beliefs Inventory (Alden and Safran, 1978)] was strongly associated with an increased 10-year risk of developing CVD. A prospective study (Wu et al., 2022) conducted on 487,209 participants, showed that continuous anxiety was positively associated with incident CVD (hazard ratio: 1.12; 95% confidence interval: 1.04–1.20) and ischemic heart disease (hazard ratio: 1.21; 95% confidence interval: 1.07–1.37).

Anxiety has been correlated to physiological arousal (Roos et al., 2021), with anxiety disorders inducing HPA axis dysfunction (Erhardt et al., 2006; Reeves et al., 2016), sympathetic hyperactivity (Richards and Bertram, 2000; Narita et al., 2007; Severino et al., 2019), endothelial dysfunction (Narita et al., 2007), increased platelet level (Almis and Aksoy, 2018), and inflammation (Michopoulos et al., 2017). Moreover, maladaptive coping increases the development of unhealthy lifestyle behaviors (Otto and Smits, 2018), thus increasing the risk factors for the development of CVD. People affected by anxiety tend to suffer from sleep deprivation (Chellappa and Aeschbach, 2022), to be scarcely engaged in physical activity (Mason et al., 2019), and more inclined to unhealthy eating and recurrent snacking (Wilkinson et al., 2013; Hussenoeder et al., 2021). People affected by anxiety are more likely to adopt smoking habits (Garey et al., 2020) and increased alcohol consumption (Torvik et al., 2019). Additionally, patients with anxiety have shown a reduction in adherence to anti-hypertensive medications (Bautista et al., 2012).

Several studies (Paterniti et al., 2001; Seldenrijk et al., 2010; Hernandez et al., 2014) have investigated how anxiety may contribute to atherosclerosis and to its progression. An observational study (Paterniti et al., 2001) involving 726 adults with no known history of CVD, showed how high levels of anxiety (assessed using the 20-item Spielberger Inventory) was associated with increased progression of atherosclerosis over a 4 year-period. A cross-sectional study (Seldenrijk et al., 2010) on 2,717 adults with depression and anxiety (assessed respectively with the Inventory of Depressive Symptomatology, and the Beck Anxiety Inventory), showed how people affected by these disorders were more likely to have subclinical atherosclerosis compared to healthy control. Another cross-sectional study (Hernandez et al., 2014) involving 1,101 middle aged adults with low CVD risk status, showed that trait anxiety (assessed with the Spielberger State-Trait Anxiety Inventory) is associated with coronary artery calcification. Dysfunction of the endothelium and consequent atherosclerosis demonstrate the potential link between anxiety and hypertension (Hernandez et al., 2014). A longitudinal study (Ginty et al., 2013) conducted in Denmark on 455 adults who were assessed for clinical depression and anxiety (using the Hospital Anxiety and Depression Scale), and followed up for 5 years, showed that patients with a diagnosis of anxiety or/and depression were more likely to develop hypertension. Furthermore, a recent meta-analysis (Emdin et al., 2016) that included 46 studies on adults with and without anxiety showed that anxiety was associated with a 41% higher risk of cardiovascular mortality and additionally reported that patients with anxiety had a 35% higher risk of heart failure, a 71% higher risk of stroke, and a 41% higher risk of coronary artery disease. The hyperactivation of the HPA axis and the increased sympathetic activity induced by anxiety and emotional stress can also lead to the onset of cardiac arrhythmias (Severino et al., 2019; Leo et al., 2023).

## Physiological and psychological effects of the hypnotic state

From a medical perspective, consciousness is a state of awareness and responsiveness to the surrounding environment, presenting two key characteristics: (i) the state of consciousness (i.e., the level of wakefulness – awake, asleep, or in coma), and (ii) the content of consciousness (i.e., the level of awareness) (Zhao et al., 2019). Studies on the neural correlates of consciousness have tried to identify the minimal set of neuronal events sufficient for a specific conscious experience, as well as identifying areas of the brains that are involved in consciousness (Demertzi et al., 2013; Calabrò et al., 2015). Several structures of the brain have been suggested having a role in consciousness, some of which are the prefrontal cortex (Pal et al., 2018) (which regulates thoughts, actions, and emotions through extensive connections with other cortical and subcortical networks), the anterior insular cortex (Medford and Critchley, 2010; Zhang et al., 2018) (which regulates functions linked to emotions and homeostasis), the cingulate cortex (Medford and Critchley, 2010; Crone et al., 2015; Herbet et al., 2016) (which is involved in emotion formation and processing), the paraventricular nucleus of the thalamus (Qin et al., 2018; Ren et al., 2018) (which is involved in sleep awakening and arousal), and the claustrum (Chau et al., 2015; White et al., 2018) (a thin sheet of neurons that connects the cerebral cortex and the subcortical areas of the brain). The latter presents a widespread connectivity to the prefrontal cortex, and to the visual, auditory, sensory, and motor regions, which suggests a controlling role over these functions to allow selective attention and prevent distraction (Goll et al., 2015).

The ‘hypnotic state’ is an altered state of consciousness, capable of modulating subjective experience (Rainville and Price, 2003) and physiological responses (Gruzelier, 1998). During hypnosis, the subject experiences an altered state of consciousness where acceptance of suggestions is facilitated. An hypnosis session includes four distinct phases (Karle and Boys, 1987; Rhue et al., 1993): (i) an induction phase, where instructions to listen to the hypnotherapist voice and to close the eyes (or fixating the eyes on a small objects – e.g., a pen) are given, (ii) a deepener phase, where suggestions of deep relaxation are given, (iii) a suggestions phase, where guided imagery is used to increase relaxation or to manage a symptom (e.g., pain reduction), (iv) an “emergence from hypnosis” phase, where instruction to come out from the hypnotic state is given.

The level of hypnotizability is related to the level in which the subject responds to the hypnotic suggestions (Rainville and Price, 2003; Vanhaudenhuyse et al., 2014), with two thirds of the world population showing to be potentially hypnotizable (Heap et al., 2004). A high level of hypnotizability seems to be associated with increased functional connectivity between the left dorsolateral prefrontal cortex and the dorsal anterior cingulate cortex (Faerman et al., 2024). Electroencephalography (EEG) data have shown that hypnotic state increases the theta band (drowsiness), with changes in the gamma band (problem solving, concentration) of brain oscillations (Vanhaudenhuyse et al., 2014; Jensen et al., 2015). A study (Jiang et al., 2016) using functional magnetic resonance (fMRI) showed reduced activity in the dorsal anterior cingulate cortex during hypnosis, with increased functional connectivity between the dorsolateral prefrontal cortex, the executive control network, and the insula in the salience network. Additionally, reduced connectivity between the executive control network, the default mode network, and the posterior cingulate cortex, was observed (Jiang et al., 2016). Another study (Vanhaudenhuyse et al., 2009) using brain imaging (fMRI) showed that the brainstem, the right primary somatosensory cortex and the left and right insula were less activated during hypnosis compared to normal wakefulness. Observation of autonomic nervous system changes during hypnosis has also shown that hypnosis reduces the tonic sympathetic nervous system activity (Aubert et al., 2009; Yüksel et al., 2013; Kekecs et al., 2016; Fernandez et al., 2021). Furthermore, it seems that hypnosis can influence HPA axis mediators (such cortisol) (Sobrinho et al., 2003; Wood et al., 2003; Goodin et al., 2012; Akgul et al., 2016; Rizkiani et al., 2021), despite the evidence being very limited.

**FIGURE 1:**
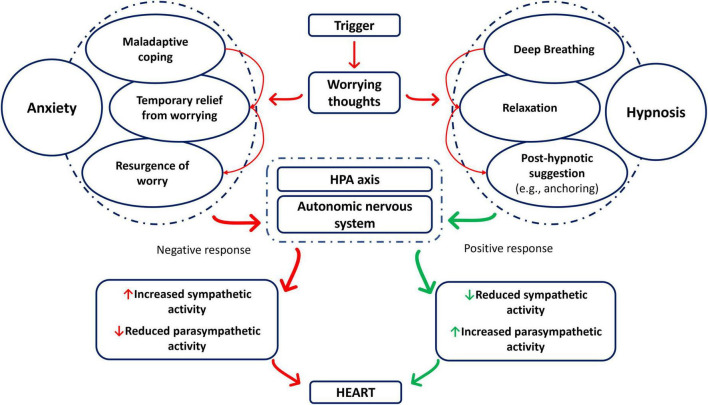
Events leading to the anxiety response, with and without hypnosis mitigation. HPA axis, hypothalamic-pituitary-adrenal axis.

The focus in obtaining a relaxing state of mind is a key aspect of hypnosis. Relaxation has shown to be effective in reducing anxiety (Kim and Kim, 2018), and relaxation techniques such as progressive muscle relaxation are often part of an hypnosis induction (Karle and Boys, 1987). A quasi-experimental study (Garvin et al., 2001) conducted on 45 healthy individuals showed that hypnosis and quiet rest are both effective in reducing state anxiety and tension. Hypnosis shows several similarities with meditation techniques (e.g., mindfulness, Yoga), both of which are effective in increasing relaxation and reducing anxiety (Bamber and Morpeth, 2019; Lemay et al., 2019). Additionally, suggestions and guided imagery during hypnosis often focus on deep breathing, to further develop a state of relaxation (Rhue et al., 1993). Deep breathing exercises have shown to be effective in reducing anxiety (Cho et al., 2016; Chen Y. F. et al., 2017), inducing reduction of the sympathetic activity and increasing parasympathetic activity (Zaccaro et al., 2018). Moreover, hypnotic ego-strengthening techniques (Rhue et al., 1993) help in coping strategies, with post-hypnotic suggestions (such as anchoring techniques) (Rhue et al., 1993) helping to break the worrying thoughts cycle that leads to anxiety (Figure 1).

Hypnotherapy, which is ‘the use of hypnosis as therapy’, focuses on inducing a ‘hypnotic state’, where the subject can receive positive suggestions aiming to positively modify their behavior (suggestion therapy) (Karle and Boys, 1987). An in-depth working model of the hypnotic process and of the neuropsychological mechanisms involved in it can be found in the review of Gruzelier (1998). Hypnosis is mostly considered a safe procedure, with clinical trials that have employed it reporting no serious adverse events (Bollinger, 2018). However, some unwanted effects may be triggered by hypnosis, that can be emotional (e.g., the recollection of a traumatic memory) or physical (e.g., headache. dizziness), and may be dependent by the hypnotic techniques used (Gruzelier, 2000). Moreover, the use of hypnosis is strongly discouraged in people suffering from paranoia, psychosis, schizophrenia, or that present borderline or dissociative characteristics (Eimer, 2012).

## “Focus on my voice”: hypnosis in the treatment of anxiety and emotional stress

A recent meta-analysis (Valentine et al., 2019) of 17 trials investigating the role of hypnosis in managing anxiety (e.g., general anxiety, performance anxiety, dentistry anxiety, medical and surgery anxiety), showed that patients treated with hypnosis achieved greater reduction in anxiety than 84% of the participants in the control group (who did not receive hypnosis). Additionally, hypnosis has shown to be even more effective in treating anxiety when combined with other psychological interventions (e.g., CBT) (Valentine et al., 2019).

A study conducted on 60 participants divided in two groups (one receiving an hypnotic safety anchor technique, and one receiving no hypnotic techniques) who were exposed to an acute stress task [Trier Social Stress Test (Kirschbaum et al., 1993)], showed that hypnosis was effective in improving stress coping. A meta-analysis (Baker et al., 2009) conducted on five randomized controlled studies showed that hypnosis is effective in reducing exam anxiety in students. Hypnosis has also shown to be effective in reducing performance anxiety in athletes. A study (Hoffmann et al., 2024) conducted on 19 downhill Mountain-bike athletes, showed that hypnosis received before competition was effective in reducing competitive anxiety and stress in the intervention group compared to a control group of other elite athletes competing in the same race. Another study (Dwivedi and Khan, 2017) conducted on 16 hockey players at University level, showed that hypnotherapy was effective in managing the symptoms of performance anxiety. A study (Chelbi, 2022) conducted on seven volleyball players showed that hypnosis was effective in reducing performance anxiety in this group.

The effectiveness of hypnosis in reducing anxiety induced by chronic diseases or medical procedures has been largely described in literature (Hammond, 2010; Mottern, 2010; Silva et al., 2022), emphasizing its positive effects and also suggesting its integration with more well-established treatment (such as CBT) (Hammond, 2010). A meta-analysis (Chen P. Y. et al., 2017) of 20 studies showed that hypnosis has an immediate positive effect on anxiety in cancer patients, with its effects being sustained in time. Similar results were shown by another meta-analysis (Sine et al., 2022) conducted on 11 studies, also showing positive effects of hypnosis on pain. A study (Schmidt et al., 2021) conducted on 31 non-invasively ventilated patients, showed that a 15 min hypnosis intervention was effective in reducing anxiety in these patients. A non-randomized controlled trial (Brugnoli et al., 2018) involving 50 patients with advanced cancer, showed that 2 years of hypnosis treatment (1 × 2 h session/weekly) had significantly decreased the pain and anxiety in the intervention group compared to standard care. The average visual analog scale (VAS) score for pain decreased from 81.9 ± 14.6 at baseline to 45.9 ± 13.8 at 1-year follow-up, and to 38.9 ± 12.4 at 2-year follow-up. Anxiety was also improved in the intervention group, with the Hamilton Anxiety Rating Scale score decreasing from 32.6 at baseline to 22.9 at 1-year follow-up and to 17.1 at 2-year follow-up (Brugnoli et al., 2018). A randomized sham-controlled crossover trial (Anlló et al., 2020), involving 21 patients with COPD, showed that a 15 min hypnosis session was effective in reducing anxiety (State-Trait Anxiety Inventory six-item questionnaire - STAI-6), which diminished highly in the intervention group (−23.8%, SD ± 18.4%) compared to control (−3.1%, SD ± 32.8). A randomized controlled trial (Courtois-Amiot et al., 2022) involving 50 older cognitively impaired patients undergoing lumbar puncture, showed that conversational hypnosis was associated with reduced self-reported (*p* < 0.05) and hetero-evaluated anxiety (*p* < 0.01), compared to controls. A double-blind randomized clinical trial (Akgul et al., 2016) involving 44 patients undergoing elective cardiac surgery, showed that hypnosis administered prior to surgery is effective in reducing pre-operative anxiety, with significant lower State-Trait-Anxiety Index-I (STAI-I) and Beck Depression Inventory (BDI) scores in the intervention group, compared to control (*p* < 0.001).

## “Breathe and relax”: effects of hypnosis on the heart

Cardiovascular reactivity is defined as a change in one or more cardiovascular parameters (e.g., blood pressure, heart rate), following exposure to a physical or psychological stressor (i.e., perceived threats to individual well-being that exceed one’s coping mechanisms) (Cinciripini, 1986). Individual reaction to psychological stressors is affected by the differences that they exhibit in their unconscious appraisal processes, elaborated by the neural circuits of the forebrain that processes internal and external sources of information, and calibrates behavioral and physiological responses (Ginty et al., 2017). Stress-evoked cardiovascular responses are a sum of the changes induced by the HPA axis activation and by the autonomic nervous system (Ginty et al., 2017). The link between cardiovascular reactivity and the onset of cardiovascular disease has been highlighted by several authors (Lovallo, 2005; Ginty et al., 2017; Silverman et al., 2019; Whittaker et al., 2021; Leo et al., 2023). Chronic sympathetic activation is known to be linked to the initiation and maintenance of hypertension, as well as to left ventricular hypertrophy, diastolic dysfunction, and atrial fibrillation (Chen et al., 2014; Lambert and Esler, 2015; Leo et al., 2023). Hypnosis has shown to be effective in regulating the autonomic nervous system by increasing the parasympathetic tone and reducing sympathetic activity (DeBenedittis et al., 1994; VandeVusse et al., 2010; Kekecs et al., 2016; Boselli et al., 2018). Autonomic cardiac tone shifts to an increased parasympathetic modulation during hypnosis (Aubert et al., 2009; Yuksel et al., 2013), possibly suggesting the beneficial role of hypnosis in cardiovascular conditions related to chronic sympathetic activation.

A randomized controlled trial (Gay, 2007) involving 30 participants with mild essential hypertension undergoing 8 × 30 min sessions of hypnosis, showed that the intervention group reduced systolic blood pressure compared to control group (standard care) after intervention (*p* < 0.003), at 6 (*P* < 0.0001) and 12-months (*P* < 0.003) follow-up. Similar efficacy was shown in the intervention group in reducing diastolic blood pressure after intervention (*P* < 0.003), at 6 (*P* < 0.001) and 12-months (*P* < 0.001) follow-up, compared to the control group. A non-randomized control study (Holdevici and Crăciun, 2013) involving 80 participants diagnosed with primary and secondary hypertension, showed that after 8-months of Ericksonian hypnosis treatment, patients in the intervention group reported higher quality of life (36-item Short Form – SF-36) post intervention (*P* < 0.05). However, due to the paucity of studies investigating the role of hypnosis in hypertension or in the management of other CVD, it is difficult to provide enough evidence to support the role of hypnosis in patients with CVD.

## Conclusions and future directions

The negative effects of anxiety in the onset and development of different CVD have been well established. Psychological stressors affect the autonomic nervous system and cardiovascular reactivity, deeply influencing the physiology of the heart. Hypnosis has been demonstrated to be effective in the treatment of anxiety disorders and of general anxiety, either alone or in combination with other psychological interventions (e.g., CBT). The altered status of consciousness induced by hypnosis has a direct effect on the parasympathetic and sympathetic activation, also influencing the autonomic cardiac tone. Hypnotic state does not only act on the management of psychological stressors (which themselves induce negative cardiovascular adaptations), but it also acts on the autonomic regulation of the heart, leading to reduced sympathetic activation, possibly suggesting protective effects toward CVD such as hypertension or atrial fibrillation. Appropriate recognition by the healthcare system on the beneficial effects of hypnosis and hypnotherapy should be acknowledged. Use of psychosocial interventions in patients at risk of developing CVD could prevent the onset of these health conditions, not only improving the quality of life and the general well-being of patients, but also reducing the costs that the National Health System (NHS) must sustain for their management.

Further research should address the current lack of studies on the role that hypnosis may play in preventing the development of health conditions affected by psychological stressors, especially on CVD. Mental health screening in the routine clinical assessment of patients at risk of developing CVD should be put in place, and hypnotherapy should assist in the management of these patients. Moreover, a deeper understanding of the neurophysiological basis of consciousness and of the hypnotic state should be further investigated, at the light of new neuroscientific discoveries.

## Limitations

Some limitations are noteworthy. The narrative nature of our review lacks a systematic approach, which may have led to an incomplete inclusion of relevant studies. Despite appropriate attention and care have been adopted during the review of the literature, some relevant research may be missing from our synthesis. Moreover, the quality of the papers in our synthesis varied markedly in methods, study design and sample size, and we did not formally assess the quality of the included studies.

## Author contributions

DGL: Conceptualization, Writing – original draft. SK: Writing – review and editing. RP: Writing – review and editing.
